# Ab Initio Calculations of the N-N Bond Dissociation for the Gas-phase RDX and HMX

**DOI:** 10.1038/srep40630

**Published:** 2017-01-17

**Authors:** Lin-lin Liu, Pei-jin Liu, Song-qi Hu, Guo-qiang He

**Affiliations:** 1 Science and Technology on Combustion, Internal Flow and Thermal-Structure Laboratory, Northwestern Polytechnical University, Xi’an 710072, P. R. China

## Abstract

NO_2_ fission is a vital factor for 1,3,5-Trinitroperhydro-1,3,5-triazine (RDX) and octahydro-1,3,5,7-tetranitro-1,3,5,7-tetrazocine (HMX) decomposition. In this study, the geometry of the gas-phase RDX and HMX molecules was optimized, and the bond order and the bond dissociation energy of the N-N bonds were examined. Moreover, the rate constants of the gas-phase RDX and HMX conformers, concerning the N-N bond dissociation, were evaluated using the microcanonical variational transition state theory (*μ*VT). The calculation results have shown that HMX is more stable than RDX in terms of the N-N bond dissociation, and the conformers stability parameters were as follows: RDXaaa < RDXaae < HMX I < HMX II. In addition, for the RDX conformers, the N-N bond of the pseudo-equatorial positioning of the nitro group was more stable than the N-N bond of the axial positioning of the nitro group, while the results were opposite in the case of the HMX conformers. Moreover, it has been shown that the dissociation rate constant of the N-N bond is influenced by the temperature significantly, thus the rate constants were much lower (<10^−10^ s^−1^) when the temperature was less than 1000 K.

Both 1,3,5-Trinitroperhydro-1,3,5-triazine (RDX) and octahydro-1,3,5,7-tetranitro-1,3,5,7-tetrazocine (HMX) represent excellent energetic nitramines. Due to their high-energy performance and smokeless, they are widely used in explosives, gun power, and solid propellants. The RDX and HMX decomposition is vital for ignition and combustion mechanism of the solid composite propellants, but the decomposition process is quite complicated because of the hundreds of involving elementary reactions^1^,^2^,^3^.

Previous theoretical and experimental studies have shown that NO_2_ fission (N-N bond fission or N-N bond dissociation), HONO elimination, concerted ring scission, and dissociation of the ring along the C–N bond are the possible initial unimolecular reaction mechanisms during the RDX and HMX decomposition process^4^,^5^,^6^. Among the listed reaction mechanisms, NO_2_ fission is considered as the vital reaction, especially at higher temperatures^7^.

Although, many of the experimental studies are devoted to the decomposition mechanisms of RDX and HMX molecules, the rate constant measurements usually provide scattered results^8^. Furthermore, the rate constant measurements are affected by several factors, such as molecular clustering and secondary reactions, therefore, the observed Arrhenius activation energy, *E*_a_, and thermal rate constants are usually unreliable^9^. Nowadays, some researchers study the NO_2_ fission of RDX and HMX molecular using the *ab initio* calculations. However, most of studies were conducted with a small basis set, which was not accurate enough for a prediction of the involved chemical reactions^10^.

Since the NO_2_ fission (N-N bond dissociation) reaction has an important role in the RDX and HMX decomposition, a theoretical study on that reaction, concerning different RDX and HMX gaseous conformers, and using the ab inito calculation is presented in this paper. The obtained results provide valuable information on the gas-phase RDX and HMX decomposition and combustion mechanisms.

## Computational details

The structural optimizations, and frequency and energy profile calculations were expedited with the Gaussian 09 program at m062x/cc-pvtz level^11^. The bonding strength was evaluated by the bond order (BO), and the mayer, wiberg and laplacian bond orders of the N-N bond for RDX and HMX were analyzed with the mutiwfn program^12^. Moreover, the bond dissociation energy (BDE) of the N-N bond strength for RDX and HMX was calculated in order to evaluate the bonding strength^13^. The BDE of A-B bond at 0 K could be defined by^14^:


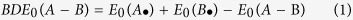


The BDE with zero-point energy (ZPE) correction can be obtained by:


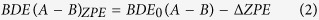


where *ΔZPE* is the ZPE difference between the products and the reactants.

Since there were no transition states in the N-N bond dissociation of RDX and HMX molecules, the rate constants of the barrierless reactions in the 400–2500 K temperature range were evaluated by the microcanonical variational theory (*μ*VT) method using the VKLab program^15^.

## Results and Discussions

### Structure of gas-phase RDX and HMX

**Figure 1:**
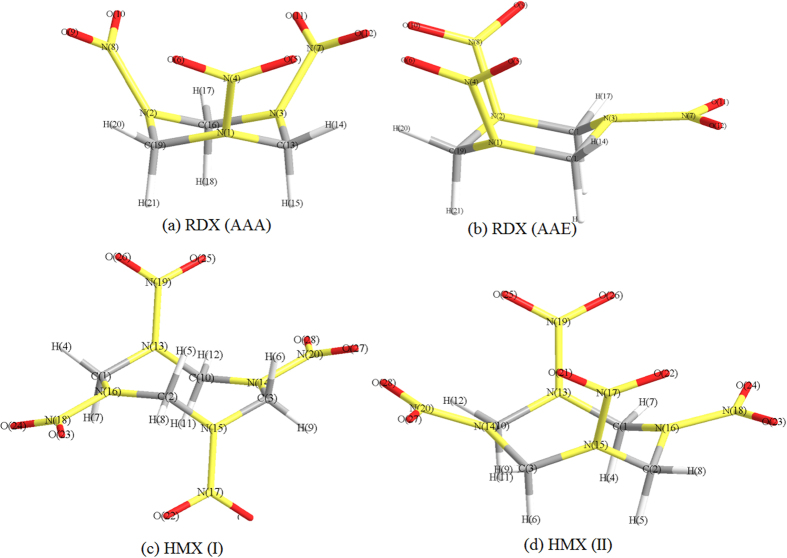
Optimized structures of RDX and HMX conformers.

There are many conformers for the gas-phase RDX and HMX, and the differences between them are mainly based on the ring shape and the nitro group position relative to the ring atoms. The RDX conformers are usually labeled according to axial (A) or pseudo-equatorial positioning (E) of the nitro groups about the ring. Some of previous works have indicated that the AAA structure, wherein all nitro groups occupy axial positions, is consistent with the electron diffraction results of the gas-phase RDX, while the AAE structure, wherein two nitro groups occupy axial positions and one nitro group occupies a pseudo-equatorial position, is consistent with the stable RDX crystal structure (*α*-RDX)^16^. In addition, some researches have shown that the N-N bond dissociation reactions are more favored for the AAA and AAE conformers. Therefore, the AAA and AAE conformers were used in this study, and the optimized structure is shown in Fig. 1. Moreover, two most stable conformers of the gas-phase HMX were examined, the first one with two nitro groups at axial positions, and the second one with two nitro groups at pseudo-equatorial positions, presented in Fig. 1(c) and (d), respectively.

The RDX conformers, presented above, belong to *C*_3V_ and *C*_s_ point group respectively, while the HMX conformers belong to *C*_2V_ and *C*_i_ point group, respectively. Therefore, according to the symmetry, there are seven different N-N bonds for the conformers, and the N-N bond dissociation for the gas-phase RDX and HMX could be studied by the investigation of these bonds properties.

### Property of N-N bonds for RDX and HMX

The calculated properties of different N-N bonds are shown in Table 1.

The results presented in Table 1 show that for RDX conformers, the N-N bond length is longer in the case of axial positioning of nitro groups. On the other hand, the results for HMX conformers are opposite, which indicates complicated properties of the N-N bonds for RDX and HMX. The bond strength usually has positive relationship with bond order, and according to results in Table 1, the bond order obtained from different methods shows the similar trend. The N-N bond of the pseudo-equatorial positioning of nitro groups has a higher bond order for RDX conformers, which indicates the higher stability of the bond. However, from the bond order aspect, the N-N bond of the axial positioning of nitro groups is slightly more stable for HMX conformers. In addition, compared with RDXaae and HMX I, the N-N bond dissociation has higher possibility to appear in RDXaae and HMX II conformers because of the lower bond orders.

The stability of the N-N bond could be also evaluated by the BDE_ZPE_. In Table 1, it is shown that BDE_ZPE_ is consistent with a bond order of some conformers, thus the same conclusions could be obtained. However, the bond order is inconsistent with BDE_ZPE_ for all conformers listed in Table 1, due to the structure difference of RDX and HMX. The BDE_ZPE_ results show that the stability of the N-N bond for RDX and HMX conformers is as follows: RDXaaa < RDXaae < HMX I < HMX II.

### Rate constant of NO_2_ fission for RDX and HMX

**Figure 2:**
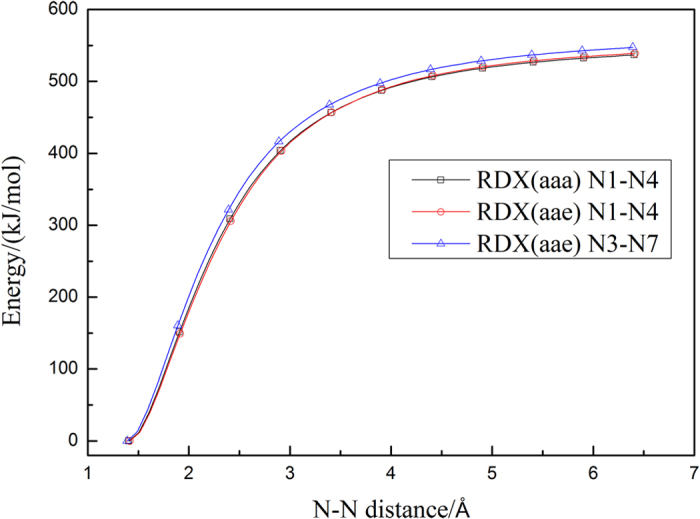
Energy profile of the N-N distance for RDX.

**Figure 3:**
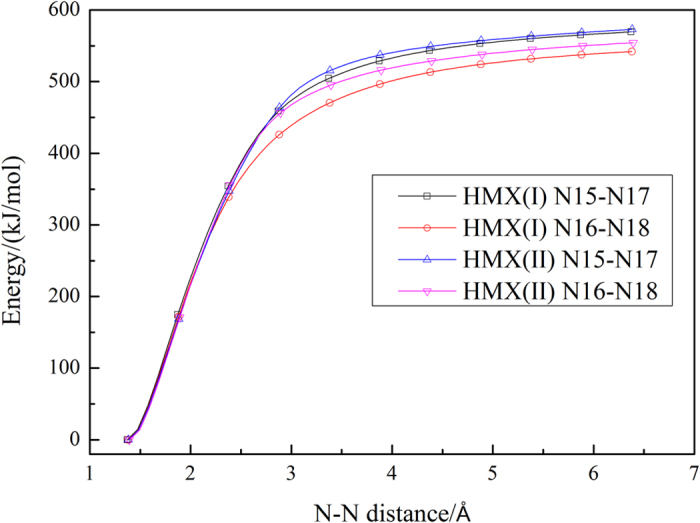
Energy profile of the N-N distance for HMX.

The potential energy along the minimum energy path (MEP) was calculated using the Gaussian09 program, and the minimum-energy profiles of the NO_2_ fission for RDX and HMX are shown in Figs 2 and 3, respectively.

**Figure 4:**
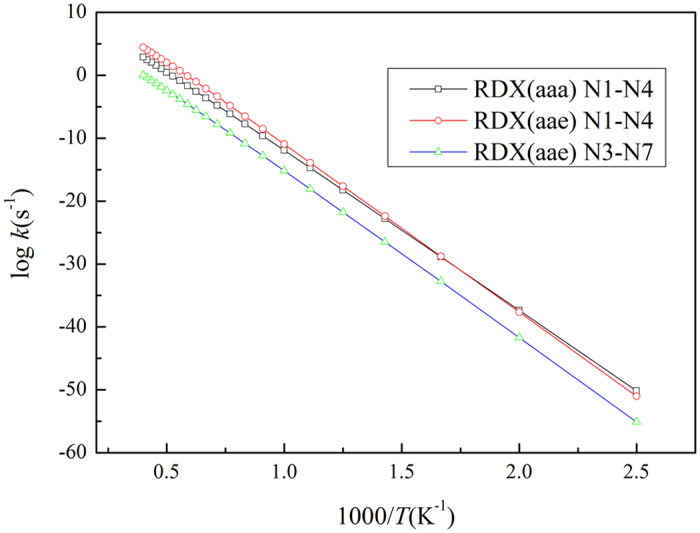
Dissociation rate constants of N-N bond for RDX.

**Figure 5:**
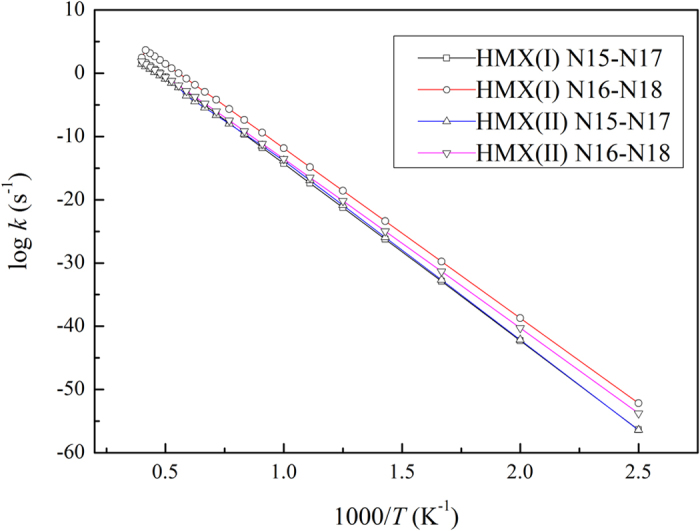
Dissociation rate constants of N-N bond for HMX.

Results presented in Figs 2 and 3, indicate that there are no transition states in the N-N bond dissociation processes. Therefore, the canonical variational transition state theory (μVT) was used to evaluate the dissociation rate constants of the N-N bond, and the obtained results are shown in Figs 4 and 5.

As it can be seen in Fig. 4, the N-N bonds of the axial positioned nitro groups for two RDX conformers have similar dissociation rate constants, and the dissociation rate constants are higher than dissociation rate constants in the pseudo-equatorial positioning cases. Nonetheless, the similar dissociation rate constants of the N-N bonds of the axial positioned nitro groups are shown in Fig. 5. As it can be seen in Fig. 5, the dissociation rate constants are lower than the dissociation rate constants in the pseudo-equatorial positioning cases. Moreover, the N-N bonds of HMX conformers are more difficult to be dissociated.

In addition, according to the relationships presented in Figs 4 and 5, the dissociation rate constants of the N-N bond are significantly influenced by the temperature, therefore the rate constants were much lower (<10^−10^ s^−1^) when the temperature was less than 1000 K. Hence, a high temperature favors the decomposition of the gas-phase RDX and HMX greatly. Moreover, the N-N bond dissociation is vital for higher reaction rates. In this study, the Arrhenius parameters were obtained by fitting of the reaction rate curves, and the results are shown in Table 2.

As shown in Table 2, the high stability of HMX, in terms of N-N bond dissociation, is consistent with the BDE_ZPE_ results. The activity energy of the N-N bond dissociation for RDX conformers was lower in the case of axial positioning of nitro groups, which indicates the higher stability of the N-N bond for pseudo-equatorial positioning of nitro groups. In the contrast to the RDX conformers, HMX conformers had higher activity energy of the N-N bond dissociation and provided more stable N-N bond in the case of axial positioning of nitro groups.

## Conclusions

According to the results presented in this paper, the following conclusions can be made:

(1) HMX conformers are more stable than RDX conformers in terms of the N-N bond dissociation due to the higher bond order, higher BDE_ZPE_ and lower reaction rate constant, and the stability of conformers is as follows: RDXaaa < RDXaae < HMX I < HMX II.

(2) For RDX conformers, the N-N bond of the pseudo-equatorial positioning of nitro groups is more stable than N-N bond of the axial positioning of nitro groups, while the results are opposite for HMX conformers.

(3) Temperature has a great impact on the dissociation rate constant of the N-N bond. The rate constants are very low (<10^−10^ s^−1^) when the temperature is less than 1000 K, thus they can be neglected. Lastly, the reaction rate is much higher at high temperature.

## Additional Information

**How to cite this article**: Liu, L.-l *et al*. Ab Initio Calculations of the N-N Bond Dissociation for the Gas-phase RDX and HMX. *Sci. Rep.* **7**, 40630; doi: 10.1038/srep40630 (2017).

**Publisher's note:** Springer Nature remains neutral with regard to jurisdictional claims in published maps and institutional affiliations.

## Figures and Tables

**Table 1:** Bond order and bond dissociation energy of N-N bond.

Bond	Bond length Å	Bond order			BDE_ZPE_ (kJ/mol)
		Mayer	Wiberg	Laplacian	
RDXaaa (N1-N4)	1.407	0.9675	1.2347	0.6087	211.66
RDXaae (N1-N4)	1.414	0.9582	1.2117	0.5829	212.95
RDXaae (N3-N7)	1.391	1.0156	1.2697	0.6789	223.47
HMX I (N15-N17)	1.373	1.0055	1.2938	0.7465	252.07
HMX I (N16-N18)	1.380	1.0232	1.2855	0.7245	219.06
HMX II (N15-N17)	1.382	0.9986	1.2694	0.7154	253.25
HMX II (N16-N18)	1.392	0.9939	1.2534	0.6492	229.82

**Table 2:** Arrhenius parameters of the reactions.

Dissociation reaction	*A*	*n*	*Ea* (kJ/mol)
RDXaaa N1-N4	4.53263E + 20	−2.16613	499.36
RDXaae N1-N4	7.04796E + 24	−3.03020	509.49
RDXaae N3-N7	1.20072E + 23	−3.54133	528.62
HMX I N15-N17	7.05727E + 21	−2.43990	551.09
HMX I N16-N18	3.91429E + 25	−3.13565	534.70
HMX II N15-N17	5.17029E + 34	−6.28984	572.74
HMX II N16-N18	4.20085E + 23	−3.13138	530.97
